# Spatial distribution of leprosy in India: an ecological study

**DOI:** 10.1186/s40249-018-0402-y

**Published:** 2018-03-27

**Authors:** Kyra H. Grantz, Winnie Chabaari, Ramolotja Kagiso Samuel, Buri Gershom, Laura Blum, Lee Worden, Sarah Ackley, Fengchen Liu, Thomas M. Lietman, Alison P. Galvani, Lalitha Prajna, Travis C. Porco

**Affiliations:** 1 Department of Biology, University of Florida, Gainesville, FL USA; 2 Emerging Pathogens Institute, University of Florida, Gainesville, FL USA; 3 DST/NRF Center for Excellence in Epidemiological Modeling and Analysis (SACEMA), Stellenbosch, South Africa; 4 Stellenbosch University, Stellenbosch, South Africa; 5 African Institute of Mathematical Sciences, Muizenberg, South Africa; 6 University of California, Berkeley, CA USA; 7 Francis I. Proctor Foundation for Research in Ophthalmology, University of California, San Francisco, CA USA; 8 Department of Ophthalmology, University of California, San Francisco, CA USA; 9 Department of Epidemiology and Biostatistics, University of California, San Francisco, CA USA; 10 Yale University, New Haven, CT USA; 11 Aravind Eye Hospital, Madurai, Tamil Nadu India

**Keywords:** Leprosy, Hansen’s disease, India, Poverty, Spatial

## Abstract

**Background:**

As leprosy elimination becomes an increasingly realistic goal, it is essential to determine the factors that contribute to its persistence. We evaluate social and economic factors as predictors of leprosy annual new case detection rates within India, where the majority of leprosy cases occur.

**Methods:**

We used correlation and linear mixed effect regressions to assess whether poverty, illiteracy, nighttime satellite radiance (an index of development), and other covariates can explain district-wise annual new case detection rate and Grade 2 disability diagnoses.

**Results:**

We find only weak evidence of an association between poverty and annual new case detection rates at the district level, though illiteracy and satellite radiance are statistically significant predictors of leprosy at the district level. We find no evidence of rapid decline over the period 2008–2015 in either new case detection or new Grade 2 disability.

**Conclusions:**

Our findings suggest a somewhat higher rate of leprosy detection, on average, in poorer districts; the overall effect is weak. The divide between leprosy case detection and true incidence of clinical leprosy complicates these results, particularly given that the detection rate is likely disproportionately lower in impoverished settings. Additional information is needed to distinguish the determinants of leprosy case detection and transmission during the elimination epoch.

## Background

Leprosy (Hansen’s Disease) is caused by a chronic infection by *Mycobacterium leprae* [1–3]. Long stigmatized in many cultures, leprosy is curable today with multidrug therapy [4]. While a concerted global effort to meet the World Health Organization (WHO) goals of elimination has greatly reduced the case burden in recent decades, over 200000 new cases are still reported globally each year [5, 6]. Current WHO targets focus on decreasing the rate of new diagnoses with Grade 2 disability, and the reversal of legislation enabling leprosy discrimination [7].

In recent years, the majority of new leprosy cases have been reported from just three countries—India, Brazil, and Indonesia. Both historically and currently, risk of leprosy infection has been linked to poverty (e.g. [8–13]). This association may arise from a combination of factors, including crowded conditions that facilitate transmission, malnutrition, or other underlying comorbidities. Though leprosy treatment is provided free of charge worldwide, the cost of travel and a lack of awareness of treatment availability may be obstacles associated with poverty to seeking or receiving health care [14].

India is uniquely important in understanding the current epidemiology of leprosy. India has had substantial success in leprosy control in previous years, but contributes over half of all global new case detections, due, in part, to its large population. Leprosy is found in all regions of the country. Furthermore, the decline in ANCDR in recent years appears to have leveled off [15–17]. Previous work has indicated that leprosy case detection in India was significantly associated with enhanced case finding activity and exhibited evidence of spatial autocorrelation [15], but it is yet unclear what factors may exacerbate leprosy burden. Here, we use publicly available district-level data on reported annual new case detection rates (ANCDR) and Grade 2 disability rates [18, 19]. We examine the association between these epidemiological outcome variables and poverty, based on other available measures of district wealth and development.

## Methods

### Data sources

#### Leprosy

The Indian Ministry of Health reports annual new case counts for leprosy for the period 2008–2015 for each district in India (see Spatial boundaries) [18–32]. In accordance with case report data, we define each year as the twelve month period ending March 31. The National Leprosy Eradication Program also provides annual estimated populations for each district, the number of new cases of Grade 2 disability (defined by the WHO as visible deformity to the hands or feet or severe visual impairment) at the district level, as well as state-level estimates for the fraction of multibacillary cases, the fraction of cases among children, and the fraction with Grade 2 disability at diagnosis.

#### Census

The 2011 Census of India contains district-level data on illiteracy, unemployment, scheduled caste and scheduled tribe populations, rural population, and poverty [33–37]. In our data set, a poverty index was defined as the absence of a defined set of assets included in the census survey. A household was considered to be impoverished in the absence of ownership of a radio, a TV, a computer (with or without internet access), a mobile phone, landline, a bicycle, or a motorized two- or four-wheel vehicle (including a scooter or car) [37, 38]. This definition is more restrictive than other economic measures of poverty (which routinely place between 20–30% of the population in poverty); only about 18% of households meet this criterion of poverty.

Illiteracy is defined as the inability to both read and write in any language; children 6 years old or younger are automatically considered illiterate in the census. An individual is considered unemployed (specifically, a “non-worker”) if he or she did not partake in an economically productive activity in the 12 months preceding the census survey. This includes students, homemakers, children, retirees, and beggars; it does not include subsistence farmers or others whose primary activity was producing food for self-consumption. Therefore, unemployment here does not necessarily indicate a desire to work, or an active pursuit of employment. The census also reports the fraction of a district’s population that lives in a rural area (defined as a region not registered as statutory town or municipality, with fewer than 5000 individuals, with greater than 75% of working individuals employed in agriculture, or with population density less than 400 per km^2^) [33].

The Constitution of India includes provisions for individuals in scheduled castes and scheduled tribes (indigenous tribal persons). Historically, these two groups experienced higher levels of discrimination, exclusion, and poverty [39]. The Census reports the number of individuals in scheduled castes and in scheduled tribes per district [35, 36], though this was not reported in 84 of the 604 analytic districts (Table 1; see Spatial boundaries, below).

**Table 1:** Data sources used in the analysis

Data	Units	Years available	Resolution	Number districts	Range	Source
Annual new case	Cases per 10,000 population	8 (2008–2015)	District	604	0–13.9	[19, 26–32]
detection rate (ANCDR)						
Grade 2 Detection	Cases per 1,000,000 population	8 (2008–2015)	District	604	0–127	[18, 20–25]
Grade 2 Fraction	Ratio	8 (2008–2015)	District	604	0–1	[18–32]
Poverty	Ratio	1 (2011)	District	604	0.01–0.65	[37]
Illiteracy	Ratio	1 (2011)	District	604	0.11–0.68	[33]
Unemployment	Ratio	1 (2011)	District	604	0.33–0.74	[34]
Scheduled Caste	Ratio	1 (2011)	District	520	0.01–0.98	[35, 36]
and Tribe population						
Rural population	Ratio	1 (2011)	District	604	0–1	[33]
Per-capita income	Rupees (fixed price)*	5 (2008–2012)	District	45	11900–55300	[41]
Visibility	–	1 (2013)	District	604	3.53–63	[46]
Radiance	–	1 (2010/2011)	District	604	0–448	[46]
Government Hospitals	Hospitals per 10,000 population	4 (2012–2015)	State	35	0.04–2.71	[40]
Per-capita net	Rupees (fixed price)*	4 (2012–2015)	State	35	22600–241000	[40]
domestic product (NDP)						

^*^Relative to 2004-2005 cost index

#### State-level predictors

While we primarily focused on district-level analysis, we examined two possible state-level predictors of leprosy burden, collected from the Centre for Monitoring Indian Economy database [40]. The first, per-capita net domestic product (NDP), is thought to be a more direct measurement of community development and wealth than poverty or other socio-demographic variables. The second, the number of government hospitals in each state, may be related to healthcare availability and accessibility.

#### Per-capita income

For validation, we compared selected indices of poverty with district-level per-capita income data. Madhya Pradesh, one of India’s largest states, reported per-capita income (held constant relative to 2004–2005 price index, thus adjusting for inflation) from 2008 to 2012 in 45 of its 48 districts [41].

#### Satellite imagery

**Fig. 1:**
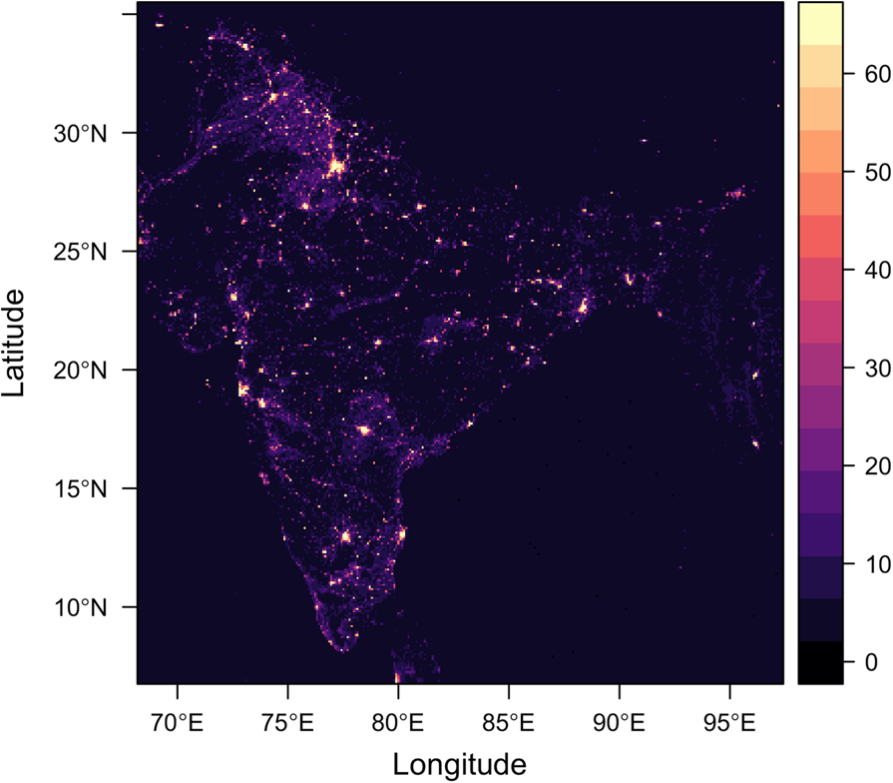
Nighttime composite satellite image. Nighttime composite satellite image of India and neighboring regions, showing average visible lights, 2013 (source: NOAA), used in regression analysis

Nighttime satellite imagery data has proven useful in assessing economic conditions in the developing world [42–45]. We obtained nighttime cloud-free composites providing average visible lights and stable lights (which excludes impermanent sources of light, such as fires or other background noise), at 30 arc second resolution (roughly 1 km; Fig. 1) [46]. In the most dense, brightly lit areas, the satellite sensors become saturated and cannot record values above a certain threshold. In India, this threshold obscures subtle differences in illumination from the country’s largest cities, including Delhi, Kolkata, Bangalore, and Mumbai. Radiance, a readjusted illumination measure produced from the same satellite imagery, may provide a better indicator of economic activity and development [44, 46]. Radiance data were derived from images taken in 2010 and 2011, and were computed by averaging radiance over the areas of each district. We computed the radiance divided by the estimated population, yielding a ratio which exhibits outliers (the largest value is approximately 14 times the average value). To minimize the occurrence of potential high-leverage points, we used the rank transformed values as a predictor. Additionally, we calculated a binary low visibility indicator, defined as 1 if a district was in the lowest decile of mean visibility index, as well as a similar low radiance indicator. No effort was made to identify oil flares or other causes of high illuminance unrelated to socioeconomic development.

#### Spatial boundaries

There were several rearrangements of state and district boundaries over the study period. Spatial analysis was based on the GADM (Global Administrative Areas) database for administrative boundaries [47], supplemented by an updated version for selected jurisdictions [48]. If a district or state was divided into multiple districts or states during the study period, we combined data from the resulting new districts to estimate what the counts would have been for the old district boundaries, to obtain a longitudinally consistent set of reporting districts and states. Likewise, if two or more regions were merged, the data from these regions was combined throughout the study period into a single analytic district. This procedure yielded 604 analytic districts from 2008–2015 (Table 1; [15]).

#### Program activities

A group of 209 districts were identified as high leprosy districts, based on 2010–2011 reports [49], and these regions were targeted for subsequent enhanced surveillance activities through the National Leprosy Eradication Program. As in our previous analysis [15], we entered this list of districts for use as a binary regressor.

### Statistical methods

#### Outcomes

The primary outcome variables were the leprosy annual new case detection rates (ANCDR), defined as the number of new cases in a district divided by the estimated population of the district during that year, and the rate of new Grade 2 disability per million population (Grade 2 rate). We also explored the heterogeneity in the proportion of reported leprosy cases that displayed Grade 2 disability (Grade 2 fraction).

We computed Spearman’s rho (*ρ*) correlation coefficients for the outcomes of interest and the potential predictors. We then conducted multivariate linear mixed effects regression [50] of the longitudinal outcomes, using the district- and state-level predictors. All models include a random slope and intercept, year as a fixed effect, and a fixed effect in 2012 and 2013 for each of the 209 enhanced case finding districts mentioned above. Spatial block bootstrap (1000 replicates) helps account for spatial dependence and often estimates a conservative confidence interval [51]. The marginal and conditional *R*^2^ values estimate the variability explained by fixed effect predictors, and by both fixed and random effects, respectively [52, 53]. To improve normality and homoskedasticity, we used the log transformation for the new case detection rates (per 10 000 inhabitants) and the per-capita Grade 2 rate (per million inhabitants), with zeros modeled as 0.5 divided by the district population (as in [15]). All analysis was conducted in R v. 3.2 for MacIntosh (R Foundation for Statistical Computing, Vienna, Austria), using packages sp, maptools, spdep, lmer, and sperrorest.

## Results

For 2008, district-level new case detection rates averaged 1.06 per 10 000 population (range 0–9.45). By 2015, the average ANCDR had decreased slightly to 0.929 per 10 000 population (range 0–13.6). While some districts reported no new cases of Grade 2 disability, others reported as many as 1.27 cases per 10 000 population, and in fifteen districts, all newly reported cases presented with Grade 2 disability. There was also substantial heterogeneity in district-level measures of poverty and development (Table 1).

Many of the covariates of interest are highly correlated with leprosy detection rates, with the two measures of Grade 2 disability frequency, and with one another. As expected, increases in ANCDR are associated with higher rates of poverty (Spearman *ρ* 0.14, *P*<0.001), illiteracy (0.20, *P*<0.001), unemployment (0.05, *P*<0.001), fraction of population in rural areas (0.12, *P*<0.001) or in scheduled castes and tribes (0.09, *P*<0.001), and the fraction of cases reported with Grade 2 disability (0.21, *P*<0.001). The population rate of Grade 2 cases is also associated with poverty (0.09, *P*<0.001), illiteracy (0.08, *P*<0.001), unemployment (-0.06, *P*<0.001), fraction of population in rural areas (0.04, *P*=0.012) and scheduled castes and tribes (0.11, *P*<0.001), and the fraction of cases reported with Grade 2 disability (0.82, *P*<0.001). Grade 2 fraction is significantly, but weakly, negatively associated with illiteracy (-0.05, *P*=0.0016), unemployment (-0.05, *P*<0.001), and rural population fraction (-0.07, *P*<0.001). Poverty and scheduled caste and tribe population are not significantly associated with the fraction of Grade 2 cases.

The raw radiance variable was strongly negatively associated with poverty (-0.68, *P*<0.001), illiteracy (-0.49, *P*<0.001), rural population (-0.64, *P*<0.001), and scheduled caste and tribe populations (-0.40, *P*<0.001). There was no significant relationship with ANCDR but a weak, positive association with rate of Grade 2 cases (0.12, *P*<0.001). Though other metrics of satellite visibility, including the scaled radiance term and visibility, were significantly associated with at least one of the three primary outcomes and other covariates, the relationships were weak and inconsistent. We therefore use the unadjusted radiance term in the remainder of our analysis.

### District-level predictors in Madhya Pradesh

We compared district-wise per-capita income in the state of Madhya Pradesh to variables under evaluation using the Spearman correlation to assess their utility in estimating economic and social conditions. Per-capita income is significantly positively correlated with satellite radiance (Spearman *ρ*= 0.57, *P*<0.001) in Madhya Pradesh. Per-capita income is also, unsurprisingly, linked to the census-derived index of poverty (*ρ*= -0.50, *P*<0.001), total visibility (*ρ*= 0.59, *P*<0.001), illiteracy (*ρ*=- 0.54, *P*<0.001) and rural population (*ρ*= -0.71, *P*<0.001). Per-capita income is not a significant correlate of unemployment or scheduled tribe and caste population. We also computed univariate Spearman regressions for the three leprosy outcomes in Madhya Pradesh, but these are small.

### District-level analysis of leprosy trends

We first computed nonparametric correlation coefficients of leprosy case detection rates with the poverty index, for every year from 2008–2015. Values ranged from 0.0686 to 0.113 (Holm-adjusted *P*-values all less than 0.012). For illiteracy, the median of these yearly correlations was 0.141 (Holm-adjusted *P*-values all less than 7.6×10^−6^). For the rural fraction, the median of these yearly correlations was 0.0805 (Holm-adjusted *P*-values all less than 0.076). Other predictors gave smaller univariate correlations (not reported).

Beginning with a base model which included the effect of time trend (year) and enhanced case finding, together with a random effect for district, we individually added each of the following predictors: (1) poverty index, (2) illiteracy fraction, (3) unemployment fraction, (4) fraction rural, (5) fraction in scheduled tribes, (6) fraction in scheduled castes, (7) log-transformed satellite radiance, and (8) the binary low visibility indicator (similar results, not shown, obtained for the binary low radiance indicator).

Illiteracy, scheduled tribe population, and radiance (including the binary low visibility indicator) are all independently significant predictors of district-level annual new case detection rate (Table 2). For the district-level rate of Grade 2 disability, the only statistically significant predictors were the fraction in scheduled tribes and the binary indicator of radiance. The fraction of cases with Grade 2 disability is significantly associated with illiteracy and unemployment rates, as well as the fraction in scheduled tribes and the binary radiance indicators. While illiteracy is a positive predictor of ANCDR, it is negatively associated with the fraction of cases with Grade 2 disability. Moreover, while scheduled tribe fraction and radiance are both negatively associated with ANCDR and Grade 2 disability rate, they are positively associated with the fraction of Grade 2 cases in each district. We also explored the role of per-capita income as a predictor of leprosy in Madhya Pradesh, incorporating a temporal dimension and random effect for district, but found non-significant relationships with all three leprosy outcomes.

**Table 2:** Regression coefficients for univariate analysis of district-level leprosy outcome variables

Covariate	Coefficient	Marginal *R*^2^	Conditional *R*^2^
Leprosy ANCDR			
Poverty	-1.69 (-5.66, 2.32)	0.05	0.68
Illiteracy	2.01 (-0.36, 6.71)	0.05	0.68
Unemployment	3.18 (-0.25, 12.6)	0.05	0.68
Scheduled Castes	4.78 (-1.29, 9.44)	0.08	0.71
Scheduled Tribes	-3.87 (-5.4, -1.41)	0.20	0.67
Rural Fraction	0.16 (-1.16, 1.64)	0.04	0.68
Radiance	0.51 (-0.05, 0.67)	0.15	0.68
Dark	-3.2 (-4.54, -1.02)	0.17	0.68
Detection rate of Grade 2 disability			
Poverty	-0.73 (-8.13, 6.61)	0.03	0.59
Illiteracy	2.21 (-2.02, 10.9)	0.03	0.59
Unemployment	0.51 (-9.58, 15)	0.03	0.59
Scheduled Castes	3.28 (-11, 14.8)	0.03	0.58
Scheduled Tribes	-5.57 (-8.41, -1.58)	0.08	0.59
Rural Fraction	-1.44 (-3, 1.67)	0.03	0.60
Radiance	0.69 (-0.17, 1)	0.06	0.59
Dark	-4.89 (-6.31, -1.01)	0.07	0.60
Fraction of cases exhibiting Grade 2 disability			
Poverty	0.04 (-0.03, 0.09)	0.02	0.21
Illiteracy	-0.04 (-0.13, -0.02)	0.01	0.21
Unemployment	-0.09 (-0.18, -0.05)	0.02	0.21
Scheduled Castes	-0.09 (-0.19, 0.005)	0.02	0.21
Scheduled Tribes	0.04 (0.007, 0.09)	0.02	0.20
Rural Fraction	− 3×10^−4^ (-0.03, 0.02)	0.01	0.21
Radiance	− 6×10^−4^ (-0.004, 0.003)	0.01	0.21
Dark	0.008 (-0.004, 0.03)	0.01	0.21

All models include calendar time in years, a covariate for the effect of enhanced case finding, a random slope, and a random intercept. Each covariate in the left hand column is separately added to the model. Confidence intervals derived by spatial block bootstrap (with a radius of 1.5 degrees; see text for details)

We then performed multivariate linear mixed effects regression to determine which covariates, in combination, produced the best-fit model (as determined by Akaike’s Information Criterion) (Table 3). We found that illiteracy, radiance, and time are included in the best models of all three leprosy outcomes. Poverty is included in the models of ANCDR and fraction of Grade 2 cases, while unemployment rate and the fraction of population that is rural are included only in the models of Grade 2 fraction and ANCDR, respectively. The coefficient for time was negative, corresponding to a (slight) decrease in ANCDR from 2008 to 2015; it appears that both the population rate and fraction of Grade 2 cases have been increasing over the same period.

**Table 3:** Regression coefficients for univariate analysis of state-level leprosy outcome variables

Outcome	ANCDR	Grade 2 Rate	Grade 2 Fraction
Poverty Index	-3.53 (-7.93, 1.12)	—	0.06 (-0.002, 0.12)
Illiteracy	4.46 (1.25, 10.3)	4.75 (0.74, 12.8)	-0.08 (-0.15, -0.03)
Unemployment	—	—	-0.06 (-0.14, -0.008)
Rural	0.9 (-0.74, 2.14)	—	—
Scaled radiance	0.01 (0.002, 0.02)	0.03 (0.02, 0.05)	2×10^−4^ (− 2×10^−5^, 2×10^−4^)
Time	-0.04 (-0.07, -0.02)	0.2 (0.09, 0.26)	0.004 (0.002, 0.005)
Marginal *R*^2^	0.04	0.01	0.02
Conditional *R*^2^	0.68	0.60	0.21

Models were selected using Akaike Information criterion (AIC) from all subsets of the regressors: poverty index, illiteracy, unemployment, rural population fraction, and scaled radiance (see text for details). All models include calendar time in years, the enhanced case finding covariate, a random slope, and a random intercept. Marginal *R*^2^ values indicate the fraction of variance explained by the fixed effects, and conditional *R*^2^ indicate the fraction of varianceexplained by both fixed and random effects as described in the text. Confidence intervals derived by spatial block bootstrap (with a radius of 1.5 degrees); see text for details

### State-level analysis

At the state level, neither net domestic product or number of government hospitals (adjusted and unadjusted for population) were significant predictors of ANCDR, Grade 2 disability rate, or Grade 2 disability fraction in univariate analysis Table 4. Healthcare availability, estimated by the frequency of government hospitals, does not appear to substantially influence reported ANCDR across states.

**Table 4:** Regression coefficients for univariate analysis of three state-level outcome variables

Covariate	Coefficient	Time trend	Marginal *R*^2^	Conditional *R*^2^
Leprosy ANCDR Per-capita NDP	-0.12	-0.003	0.0034	0.964
Hospitals (per 10 000)	-0.26	0.004	0.005	0.326
Hospitals	3×10^−4^	0.01	0.016	0.354
Grade 2 new diagnosis rate				
Per-capita NDP	-1.65	0.58	0.045	0.586
Hospitals (per 10 000)	-0.07	0.07	1.2×10^−4^	0.803
Hospitals	0.002	0.18	0.08	0.804
Fraction Grade 2				
Per-capita NDP	-0.69	0.85	0.017	0.733
Hospitals (per 10 000)	0.18	0.42	0.0026	0.724
Hospitals	-0.001	0.38	0.017	0.721

We show results for state-level new case detection rates, Grade 2 disability detection rate, and fraction of cases displaying Grade 2 disability. All models include calendar time in years, a random slope, and a random intercept. Marginal *R*^2^ values indicate the fraction of variance explained by the fixed effects, and conditional *R*^2^ indicate the fraction of variance explained by both fixed and random effects; see text

## Discussion

Leprosy incidence has decreased dramatically in recent years, spurred by ambitious WHO goals for elimination and by concerted effort by many of the most affected countries. Nonetheless, uncertainty remains regarding the factors underlying its persistence in certain geographic regions. Here, we examined the role of poverty and other measures of socioeconomic status in explaining variation in the district-level new case detection rates of leprosy in India. Modest relationships between leprosy annual new case detection rates and a census-derived poverty index of poverty were seen in univariate analysis. Higher rates of illiteracy were associated with a higher ANCDR but a lower fraction of Grade 2 cases; the inverse is true of the scheduled tribe population fraction, which is negatively correlated with ANCDR and Grade 2 detection rate, but positively associated with the fraction of Grade 2 cases. Other variables (unemployment, scheduled caste population, rural population) yielded nonsignificant relationships.

The unadjusted radiance term is a significant predictor of higher ANCDR in both univariate and multivariate analysis. However, the binary variable indicating whether a district is in the darkest 10% of all districts is significantly negatively associated with ANCDR. Considerable reporting heterogeneity between states or districts may make rates difficult to compare between regions, and may be associated with many of the covariates studied here. The poorest districts may have less capacity for detection and surveillance, resulting in lower ANCDRs than would be expected. A number of independent reports have also indicated that leprosy incidence might be considerably higher than reported incidence, and that many cases continue to go undetected by national surveillance systems [16, 54, 55]. Beyond our finding of a modest decrease in the detection rate of new leprosy cases from 2008–2015 (consistent with a previous study [15]), we also found a slight increase in the rate of detection of Grade 2 cases and the fraction of detected cases presenting with Grade 2 disability. Others have noted a similar increase or stability in the rate and fraction of Grade 2 cases, even in the event of an overall reduction in leprosy burden [56–59].

Those factors that were significantly positively predictive of ANCDR were all significant negative predictors of the fraction of Grade 2 cases in univariate analysis (Table 2). Again, reporting capacity may be a confounding factor. While there could be a true increase in the incidence of Grade 2 disability relative to the number of new leprosy cases, it is also possible that districts with higher ANCDR have better surveillance or reporting systems, finding cases before they progress to Grade 2. Conversely, districts with less detection or reporting capacity (and consequently, lower ANCDR) could be more likely to detect mostly severe, Grade 2 cases. Poverty may both increase exposure to conditions favoring the transmission of disease as well as reduce detection and reporting.

Several limitations apply to this analysis. As discussed above, ANCDR does not perfectly reflect true leprosy incidence. Moreover, the use of the census-derived poverty index and satellite radiance do not fully characterize poverty. While these covariates were strongly correlated with per-capita income in one state, many other aspects of poverty, healthcare availability, and development status may be important drivers of leprosy persistence. Our analysis is also limited due to its ecological nature; from these data, it is impossible to ascertain the relationship between poverty and leprosy within a district or at an individual-level. Furthermore, several determinants of leprosy persistence may be manifested on a geographic scale smaller than that studied here. There is some evidence that leprosy occurs in relatively small spatial clusters (even within districts) [17, 59–61]. Analysis at a finer spatial scale may be needed to more definitively identify the key drivers of leprosy transmission and case detection.

## Conclusion

We found evidence of a modest relationship between poverty and leprosy at the district level for India, in the context of a slowly declining incidence. Our results also emphasize the role of surveillance capacity in the detection, treatment, and prevention of leprosy cases—indeed, a large scale population-based detection campaign has been recently undertaken across endemic districts [62]. More information at the individual level, from cross-sectional population-based surveys and assessment of surveillance capacity, is needed to understand the relationship between poverty and leprosy, and to overcome poverty and stigma as obstacles to leprosy elimination.
